# THE ROLE OF LIFESTYLES IN ETHNIC DIFFERENCES IN COGNITIVE IMPAIRMENT AMONG ADULTS AGED 50 YEARS OR OLDER

**DOI:** 10.1093/geroni/igad104.2958

**Published:** 2023-12-21

## Abstract

**Background:**

We examined ethnic differences in the prevalence of cognitive impairment and explored the impact of lifestyles on disparities in cognitive function among adults aged 50 years or above in Western China.

**Methods:**

6,840 participants aged 50 years or older from the West China Health and Aging Trend Study (WCHAT) were included. Cognitive impairment was identified by the Short Portable Mental Status Questionnaire (SPMSQ). We used logistic regression to examine the association between ethnicity (seven groups) and cognitive impairment and the contribution of lifestyle factors to ethnic disparities.

**Results:**

The prevalence of cognitive impairment ranged from 6.2% in Bai to 29.3% in Yi. After multivariable adjustment, Tibetan had the highest odds of cognitive Impairment (OR=3.22, 95% CI 2.55-4.08), followed by Yi (OR=2.76, 95% CI: 2.121-3.59), Uighur (OR=2.59, 95% CI: 1.86-3.61) and Qiang (OR=2.10, 95% CI: 1.67-2.65), compared to Han. Lifestyle factors explained 22.2% of the ethnic difference in cognitive impairment.

**Conclusions:**

The prevalence of cognitive impairment varied widely across ethnic groups. Interventions promoting healthy lifestyles might help reduce the ethnic disparities in cognitive function among Chinese older adults.

